# A Review on Catalytic Fast Co-Pyrolysis Using Analytical Py-GC/MS

**DOI:** 10.3390/molecules28052313

**Published:** 2023-03-02

**Authors:** Sabah Mariyam, Shifa Zuhara, Prakash Parthasarathy, Gordon McKay

**Affiliations:** Division of Sustainable Development, College of Science and Engineering, Hamad Bin Khalifa University, Qatar Foundation, Doha P.O. Box 34110, Qatar

**Keywords:** co-pyrolysis, catalyst, py-GC/MS, bio-oil, aromatics, zeolite

## Abstract

Py-GC/MS combines pyrolysis with analytical tools of gas chromatography (GC) and mass spectrometry (MS) and is a quick and highly effective method to analyse the volatiles generated from small amounts of feeds. The review focuses on using zeolites and other catalysts in the fast co-pyrolysis of various feedstocks, including biomass wastes (plants and animals) and municipal waste materials, to improve the yield of specific volatile products. The utilisation of zeolite catalysts, including HZSM-5 and nMFI, results in a synergistic reduction of oxygen and an increase in the hydrocarbon content of pyrolysis products. The literature works also indicate HZSM-5 produced the most bio-oil and had the least coke deposition among the zeolites tested. Other catalysts, such as metals and metal oxides, and feedstocks that act as catalysts (self-catalysis), such as red mud and oil shale, are also discussed in the review. Combining catalysts, such as metal oxides and HZSM-5, further improves the yields of aromatics during co-pyrolysis. The review highlights the need for further research on the kinetics of the processes, optimisation of feed-to-catalyst ratios, and stability of catalysts and products.

## 1. Introduction

Recently, there has been growing interest in utilising sustainable energy resources, such as biomass and biofuels, to decrease dependence on fossil fuels and curb climate change. These types of renewable energy can be derived from organic materials, including plants and wood, and can significantly decrease the emission of greenhouse gases (GHG). [1]. Biomass co-pyrolysis has gained attention as a potential method to produce bio-oil, which can be used as a renewable energy source. Bio-oil is produced through the thermal decomposition of organic wastes under oxygen-limited conditions. When biomass is co-pyrolysed with another carbonaceous material, such as coal or plastic, the resulting bio-oil can have improved properties compared to bio-oil produced from biomass alone [2,3]. However, challenges still need to be addressed to optimise the process, including the need for a more efficient conversion of biomass to bio-oil and improving the quality and stability of the bio-oil produced [4].

Catalytic fast co-pyrolysis has gained significant attention in recent years as an effective and sustainable method for producing biofuels and chemicals while reducing waste disposal and using cost-efficient, non-edible feedstocks while maintaining high levels of energy in the resulting liquid products, up to 70% [5,6]. The process involves the simultaneous pyrolysis of biomass in the presence of a catalyst at high temperatures, producing bio-oils and gases (syngas) with enhanced yields and quality [7]. Analysing the products and intermediates produced during catalytic biomass fast co-pyrolysis using pyrolysis-gas chromatography-mass spectrometry (Py-GC/MS) is a reliable and efficient method.

This technique combines analytical pyrolysis (Py) with gas chromatography (GC) and mass spectrometry (MS) and can quickly and effectively analyse the results with minimal sample requirements [8,9]. Py-GC-MS is an effective analytical method with multiple benefits in the assessment of intricate mixtures. One of its primary strengths is its capability to determine the products during a single experiment without the requirement of preliminary separation [10]. Consequently, the method is notably efficient, and time-saving compared to other separation methods. Additionally, it can analyse both liquid and solid samples, including solid polymers that have been dissolved in an appropriate solvent. The procedure is highly automated, reducing the analysis time and improving productivity. Py-GC-MS is also highly sensitive, detecting concentrations as low as 50 mg OPA/kg. Finally, the technique provides a wealth of data regarding the analytes’ molecular structures and elemental compositions, which makes it an invaluable tool in fields such as material science and forensics. This review paper intends to analyse the application of utilising the technology to analyse the volatiles produced during co-pyrolysis.

Using catalysts in fast biomass co-pyrolysis improves the yield and quality of the bio-oil and syngas. The choice of catalyst is crucial, as it plays a significant role in determining the reaction pathways and the types of products formed [11]. Noble metals, such as platinum (Pt) and palladium (Pd), have been widely used as catalysts in biomass-fast co-pyrolysis due to their high activity and selectivity [12]. However, their high cost and limited availability have led to the exploration of alternative catalysts, including transition metals, metal oxides, and zeolites [12].

Various articles focus on fast co-pyrolysis by utilising unique waste feeds such as macroalgae with plastics [13], printed circuit boards, waste tires [14], and Naomaohu coal and cedar mixture [15]. Considering the increasing interest in the subject, few recent review publications have attempted to understand fast pyrolysis from co-pyrolysis. While Zhang et al. focused on co-pyrolysis of lignocellulosic biomass and polymers [5], Zhong et al. examined the upgrading of lignocellulosic biomass for bio-oil application via increasing aromatic hydrocarbons (AHs) [16]. Others have centered their research on the catalysts: for example, a recent article by Mishra et al. focused on the effect of the acidity, structure and porosity of zeolites during the pyrolysis of single biomasses [17]. Additionally, Shahdan et al. assessed the utilisation of metal-modified HZSM-5 for the co-pyrolysis of agricultural residues and plastic wastes. 

The novelty of this study relies on multiple aspects: (i) it focuses on the studies published utilising Py-GC/MS for catalytic biomass fast co-pyrolysis; (ii) it attempts to understand the reaction mechanisms when biomasses are mixed with biomasses, plastics, or other rare feeds [18]; (iii) it also discusses the effect of zeolites (single and in combination with others), as well as rare earth catalysts, including the catalytic behavior of the feeds during co-pyrolysis. To the best of the authors’ knowledge, this is the first paper that has attempted to focus on the above objectives. 

The fact that Py-GC-MS allows for the identification and quantification of the products and intermediates produced during the reaction and determining their chemical structures is critical in optimising the process and developing new catalysts and reaction conditions. For the co-pyrolysis of feeds for bio-oil production, various other factors influence the performance, including the type of biomass and catalyst and their ratios, the reaction temperature, and heating rates [19]. Optimising these parameters is essential to achieve the desired product yield and quality. Hence, this review aims to present an updated understanding of the latest developments in catalytic biomass fast co-pyrolysis using Py-GC/MS. It focusses on the progress that has been made and the limitations/challenges that are associated with the process. This critique also discusses the various catalysts, reaction conditions, and the products and intermediates produced during the reaction. Therefore, the objectives of this review are as follows:

- To review bio-oil analysis from fast catalytic co-pyrolysis using a micro-pyrolyser attached with GC-MS.
- To discuss the effect of feedstock and catalyst type on product generation of volatiles.
- To understand commonly proposed reaction mechanisms due to catalytic co-pyrolysis of biomass with various feedstocks.
- To discuss how to increase specific volatile product yields by altering feedstock and catalyst ratios.
- To suggest the research gaps and opportunities for future work.

**Figure 1:**
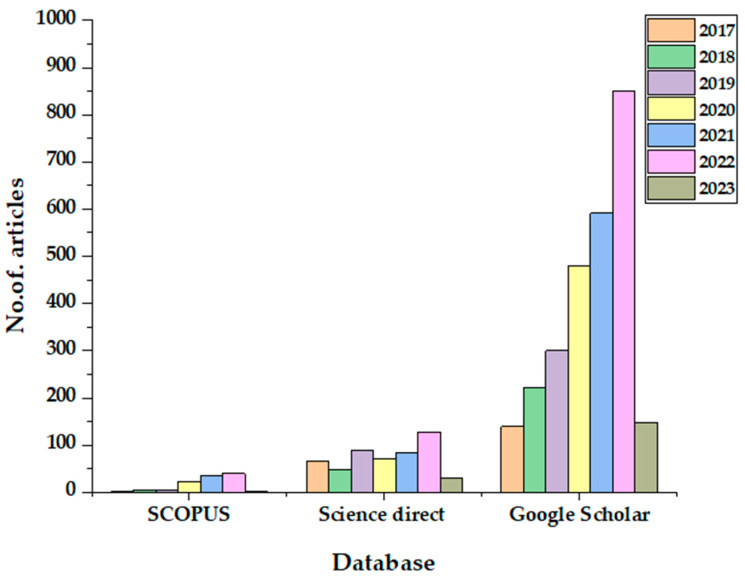
Trends in Py/GC-MS based co-pyrolysis studies in various databases.

**Figure 2:**
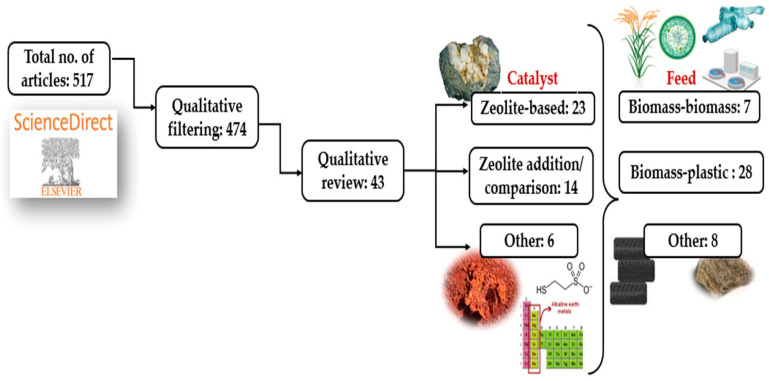
A graphical illustration of the methodology of this review article.

A literature survey has been undertaken to understand the increasing trend in the articles published on the fast co-pyrolysis of biomass and wastes. The keywords “analytical” and “py-gc/ms” and “catalytic” and “co-pyrolysis” and “biomass” were entered into three databases—SCOPUS, ScienceDirect and Google Scholar—to generate the results. Figure 1 shows the increasing trend of publications in the field and the maximum and minimum results, which were derived from Google Scholar and SCOPUS databases. Since the databases over- and under-generated the results, the authors qualitatively filtered the articles from ScienceDirect from 2017 to 2023 on the 25th of December 2022. Figure 2 shows the methodology of the literature selection of this review article; a total of 43 articles were selected, ensuring the main objectives of utilising Py/GC-MS for catalytic co-pyrolysis. Section 2 of this study discusses zeolite catalysts (single or comparison) studies divided based on feedstocks into biomass with biomass (Section 2.1), biomass with plastics (Section 2.2 and other feeds (Section 2.3). Section 3 discusses zeolites combined with other catalysts following the same subsections as Section 2. Section 4 discusses catalysts other than zeolites. Finally, Section 5 summarizes the discussed studies and identifies research gaps. Section 6 concludes this review article.

## 2. Zeolite Catalysts—Single or Comparison

Zeolites are “crystalline aluminosilicates with a tetrahedral framework structure enclosing cavities occupied by cations and water molecules, both of which have enough freedom of movement to permit cation exchange and reversible dehydration” [20]. They have undergone rapid and dynamic advancements since their introduction to petroleum processing in the 1960s [21]. Due to their unique pore structure and acidity, they are considered efficient catalysts for deoxygenation and cracking. 

Several zeolites, including micropore zeolites (H-Y, HZSM-5, and H-) and mesopore zeolites (USY, MCM, and SAPO), have been used as catalysts [22]. They have been proven to improve the properties and quality of liquid products, making them more suitable for biofuel purification. Because of their high selectivity towards valuable hydrocarbons, high acidity, and excellent hydrothermal stability, zeolites have also been used for catalytic pyrolysis of feedstocks such as lignocellulosic biomass, plastics, and so on. To top it all, they are relatively inexpensive. Zeolites for catalyst applications typically cost between 3 and 4 per USD/kg [23]. A plethora of studies have confirmed that adding zeolites as catalysts in pyrolysis optimises the reaction behavior and influences product distribution. The usage of zeolites as a catalyst in the fast catalytic co-pyrolysis of biomass-biomass feedstocks, biomass-plastic feedstocks, and biomass-other feedstocks has been discussed in the following section.

### 2.1. Biomass-Biomass with Single Zeolites

MSW components—PVC, sawdust, cotton fabric, paperboard, and vegetables—were previously proposed as useful feed inputs for the generation of value-added products. While PVC produced more benezoid compounds, sawdust and vegetables produced higher acids [24]. This article reviews the work of He et al. (2021) on the synergistic deoxidation effect of municipal solid waste (MSW) and corn stalk co-pyrolysis using ZSM-5 and nMFI zeolites [25]. Using the catalysts, the pyrolysis product compositions differed from those obtained without adding a catalyst. The oxygenated organics produced by co-pyrolysis of feedstocks without catalysts were reduced by −1.2% to −14.6% compared to the theoretical value. It also increased the hydrocarbon content of the pyrolysis products, but it drastically reduced the residual weight of the feedstocks. Zeolite catalysts aided in the breakdown of long-chain olefins, resulting in the formation of short-chain olefins. Co-pyrolysis with ZSM-5 converted feedstock into AHs via olefin cyclization and aromatization, yielding up to 58% AHs. In comparison to ZSM-5, nMFI with more Lewis acid sites induced the Diels–Alder reaction, forming more furans. The study also confirmed that corn stalk and MSW co-pyrolysis had a synergistic deoxidation effect.

An analysis performed by Chaerusani et al. (2022) focused on the effects of biomass types on the upgrading of bio-oils produced with zeolite catalysts [26]. In this study, biomasses such as rice husk, cedar, and marine eelgrass were analysed, as well as various commercial zeolites such as H-ZSM-5 (H-ZSM-5–40; Si/Al 40), H-USY (H-USY-6; Si/Al 6), H-Mordenite (H-MOR-15; Si/Al 15), H-Beta (H-BETA-40; Si/Al 40), and H-Ferrierite (H-FER-18; Si/Al 18). The findings demonstrated that the compositions of bio-oils are significantly influenced by the biomass type, as evidenced by the compositions of the bio-oils. For instance, the cedar bio-oil contained acid compounds, phenols, and ketones, but no AHs, while no phenol was found in the eelgrass bio-oil. Toluene, benzene, and xylene, on the other hand, were found in all of the biomass bio-oils. The H-ZSM-5-40 produced the highest bio-oil yield out of all biomasses compared to H-USY-6 and H-BETA-40 catalysts. This is because of its high steric hindrance (CI index-6.9) and low pH values (high acidity). It also produced the most AHs (77%) in the bio-oils. Additionally, for all feedstocks, the H-ZSM-5-40 catalyst was found to have the least amount of coke deposition. During the pyrolysis process, various gases such as H_2_, CH_4_, CO, and CO_2_ were also synthesised. CO was the primary compound of gas products. Among the feedstocks, eelgrass yielded the highest gas production. 

*Chlorella* have been known to produce about 25.6% aromatics and 21.64% nitrogen-based compounds [27]. This study reviews the work of Li et al. (2021), who investigated the co-pyrolytic thermal behavior of MSW, *Chlorella vulgaris*, and their mixtures using a ZSM-5 catalyst [28]. The catalyst influence on the composition of pyrolysis products such as AHs, N-compounds, acids, furans, and saccharides was also compared to the effect of the other three zeolite frameworks consisting of ZSM-5, Al-SBA-15, and Al-MCM-41. The study also looked at the characteristics of coking with hierarchical ZSM-5 zeolite. The co-pyrolysis of feedstocks showed considerable synergistic effects between 260 and 330 °C at a 5:5 feedstock-to-catalyst ratio of 5:5. All four zeolites significantly reduced the residual weight of the feedstocks. The residual weight of the feedstocks without zeolites was 22%, but after catalytic pyrolysis, it decreased to 18–21%. The AH study established that Hi-ZSM-5 possessed excellent catalytic attributes for complex reactants in decarboxylation and aromatization. Meanwhile, the experimental results showed that hierarchical ZSM-5 (with micropores and mesopores) exhibited excellent mono-AH selectivity (34%) but poor acid selectivity (9%).

### 2.2. Biomass-Plastics with Single Zeolites

Zhang et al. (2021) investigated the behavior of co-pyrolysis products of chili straw and polypropylene (PP) under a variety of operating conditions, including feedstock blending ratio, catalyst addition, and microwave pre-treatment [29]. This research made use of the catalyst HZSM-5. The bio-oil was characterized using GC-MS by analysing components such as olefins, alkenes, AHs, oxygen compositions, and others. The results showed that adding the catalyst increased the AHs content of the product from 4.46% to 17.34%. Furthermore, the conversion efficiency of raw materials to bio-oil improved, as evidenced by a decrease in final residual content (12.75% to 7.71%). According to the findings, the addition of the catalyst positively affected the co-pyrolysis process.

Mullen et al. (2018) focused on studying the co-pyrolysis behavior of polyethylene (PE) and switchgrass in the presence of an HZSM-5 catalyst using Py-GC/MS [30]. The effect of co-pyrolysis on coke formation and HZSM-5 deactivation was analysed using different catalyst ratios. Co-pyrolysis over fresh catalyst produced olefins and furans, which combined to produce AHs while reducing coke formation. The reduced coke formation resulted in a reduction in the initial rate of HZSM-5 deactivation by a factor of up to 2:1 at the beginning of the process. However, as more pyrolysis vapors were exposed to the catalyst, the influence of blending on deactivation decreased.

In this study, Huang et al. (2022) investigated the synergistic interaction of low-density polyethylene (LDPE) with biomass model constituents (cellulose (CE), xylan, and lignin) over hierarchical HZSM-5 [31]. The synergistic interaction between LDPE and biomass model constituents was in the following order: CE-LDPE > xylan-LDPE > lignin-LDPE. Furan yields were higher from CE than from xylan or lignin. Furan synthesis played a crucial synergistic role in the Diels–Alder reaction that produced light AHs. The main synergistic interaction that significantly increased the AH yield was the Diels–Alder reaction between olefins and furans. The synergistic effects of mixing cellulose with plastics is an improvement to a previous study that found that adding ZSM-5 to cellulose reduced the furan content considerably [32]. 

**Figure 3:**
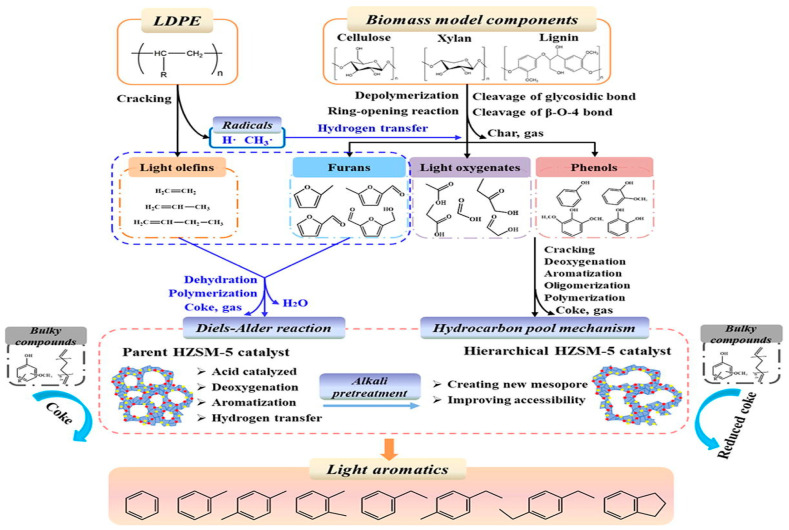
Possible synergistic reaction pathways during co-pyrolysis of biomass components with LDPE over HZSM-5 [31]. Copyright 2022, Elsevier.

Figure 3 depicts the reaction mechanisms of the co-pyrolysis over the HZSM-5 catalyst outlining the Diels–Alder reaction, hydrocarbon pool mechanism, and the alkali pretreatment in generating light olefins, furans, light oxygenates, phenols, and light AHs.

An investigation of co-pyrolysis studies of PE and lignin employing an HZSM-5 catalyst was conducted by Ke et al. (2022) using a Py-GC/MS analyser [33]. The results illustrate that the co-pyrolysis process yields benzene, toluene, ethylbenzene, and xylene (BTEX). With the following conditions: 650 °C, 0.5 MPa pressure, 1:1 PE/lignin ratio, and 4:1 catalyst/raw material ratio, an extremely high BTEX yield of 71% was obtained. Benzene and toluene have low CI values, so they are more likely to reduce and poly-condensate into AHs.

Using TGA and Py-GC/MS (HZSM-5) analysers, Zheng et al. (2018) investigated co-pyrolysis of biomass samples (pine sawdust and CE) and LDPE with (HZSM-5) and without a catalyst [34]. The study’s objective was to increase the selectivity and yields of AHs in biomass catalytic pyrolysis by incorporating LDPE. TGA results showed that biomass and LDPE interact synergistically. Biomass-LDPE co-pyrolysis increased the AH yield and selectivity while decreasing the selectivity for BTEX. In all feedstocks, the catalyst increased syngas yield. Furthermore, it resulted in a reduction in bio-oil production. These findings indicate that CE decomposes faster and that the HZSM-5 catalyst is highly effective in pyrolysis cracking. The presence of a catalyst increased the efficiency of producing AHs.

Zhao et al. (2020) investigated the synergy of CE and PE co-pyrolysis over HZSM-5, as well as the mechanism of catalytic co-pyrolysis [35]. The Py-GC/MS results revealed that the co-pyrolysis of feedstocks and the addition of HZSM-5 catalyst significantly reduced the oxygenates’ composition in the pyrolysis products (from 93 to 28%) while increasing the content of ketones, esters, AHs, olefins, and alkanes. HZSM-5 increased the AHs produced by the Diels–Alder reaction.

Using two catalysts (HY and HZSM-5), Park et al. (2019) evaluated the co-pyrolysis of waste plastic films and *Quercus variabilis* for its degradation characteristics [36]. The TGA results demonstrated a robust interaction between waste films and *Q. variabilis* pyrolysis intermediates, while Py-GC/MS characterisation studies showed that in the absence of the catalysts, the typical co-pyrolysis products (pyrolysates) of feedstocks did not change. However, when waste film and *Q. variabilis* pyrolysis intermediates were co-pyrolysed in the presence of catalysts, the interaction resulted in additional AH synthesis. The catalytic pyrolysis of feedstocks primarily produced light hydrocarbons, CO, CO_2_, and AHs (BTEX, naphthalene, and alkyl naphthalene). Compared to HY, HZSM-5 produced a higher yield of AHs while having a lower coke formation efficiency. This is because HZSM-5 has an appropriate pore size and a high acidity. Comparing two mesoporous catalysts over linear low-density polyethylene (LLDPE) and biomass carbohydrates (xylan, avicel (CE), and torrified avicel) revealed that the strong acidity and the presence of both micropores and mesopores of MZSM-5 produced more mono-AHs rather than by the presence of Al-SBA-15 [37]. Additionally, the effect of the torrefaction of avicel increased furan production due to structural changes. The Diels–Alder reaction yielded better AH production during the co-pyrolysis. 

Using Py-GC/MS, Xiaona et al. (2019) investigated the catalytic pyrolysis of PP and poplar wood composite over four distinct zeolite catalyst types (HBeta, HY, HZSM-5, and HUSY) [38]. The study’s focus was to see how the above zeolite types affect both the distribution of pyrolysis products and the feedstocks’ combined effect. The findings demonstrated that both the acidity and pore size of zeolites significantly influenced the pyrolysis products’ composition and produced a synergistic effect. According to the study, the AH production in zeolites was in the following order: HZSM-5 > HY > HBeta > HUSY. The difference in AH generation is due to differences in the acidities and pore size of the zeolite frameworks. The zeolites HBeta and HY improved the poly-alkyl aromatic selectivity, whereas HZSM-5 bettered the formation of AHs. In terms of coke production, HZSM-5 displayed the lowest yield (6%). The study concluded that by catalytically pyrolysing poplar wood and PP composite, value-added hydrocarbons can be produced through the use of zeolite catalysts. Similar findings due to the effect of acid sites on the catalysts have been reported in other studies of biomass and PP with various zeolites, including MCM-41 (modified and unmodified), and are mentioned in Table 1 [39,40,41]. Additionally, the acidic sites and pore types of MFI nanosheets have been reported to improve the mono-AH and BTEX yields during the co-pyrolysis of water hyacinth and scrap tires [42]. 

Sarkar et al. (2020) used a Py-GC/MS analyser to co-pyrolyse high-density polyethylene (HDPE) and poplar wood sawdust over acid-treated ZSM-5 [43]. The study’s goal was to improve hydrocarbon formation in the pyrolytic vapour. The ZSM-5 was acid modified with H_2_SO_4_ using a wet impregnation method. The results showed that acid treatment changes the number of acid sites in ZSM-5, influencing catalytic activity. Co-pyrolysis (without catalysts) produced a higher olefin yield (53%) than individual poplar pyrolysis (17%). It also significantly reduced the amount of oxygenates. In the case of co-pyrolysis of feedstocks with acid-modified ZSM-5, the olefin content improved and ranged between 56% and 60%, whereas the parent P-ZSM-5 contributed 50% olefin. Acid-modified ZSM-5 also produced more alkanes than P-ZSM-5 and was more selective to AH formation. In terms of deoxygenation capacity, acid-treated catalysts performed better than the parent catalyst. The study concluded that ZSM-5 could be used to co-pyrolyse plastic and biomass to generate high-quality bio-oil.

### 2.3. Other Feeds with Single Zeolites

Chen et al. (2019) used TGA Fourier-transform infrared spectroscopy (FTIR) and Py-GC/MS analysers to investigate the characteristics of tire and kitchen waste co-pyrolysis [44]. TGA-FTIR results revealed that CO, CO_2_, SO_2_, C-H, C=C, NO, and NH_3_ are major gas elements that were released from the process. The co-pyrolysis of the two feedstocks demonstrated a positive influence on the degradation kinetics, confirming that the co-pyrolysis accelerates the process’s reactivity. The Py-GC/MS outcomes showed the process produces mono-AHs, poly-AHs, olefins, organic carbon, and others. It was observed that co-pyrolysis increased volatile, and olefin yields but hindered non-hydrocarbon compound synthesis.

Using a Py-GC/MS analyser over HZSM-5 zeolite, Fan et al. (2020) co-pyrolysed lignin samples with waste cooking oil to produce AH compounds [45]. The study’s main objective was to valorise lignin residue and cooking oil waste. As model samples, the mechanism of AH formation was also studied using lignin-based monomers such as o-cresol, phenol, and guaiacol. The study also looked at the effect of adding cooking oil to lignin samples, as well as the effect of HZSM-5 on products’ output and selectivity. A previous study found the pyrolysis of waste cooking oil (palm oil) produced unsaturated, saturated, aromatic hydrocarbons, carbonyl, and oxygenated compounds [46]. The catalytic co-pyrolysis yielded bio-oil-containing organic compounds such as mono-AHs, aliphatics, poly-AHs, fatty acids, phenols, and oxygenates, including aldehydes, acids, and ketones. According to the findings, a high percentage of cooking oil in the feedstock mixture and an ideal catalyst-to-feedstock ratio (3:1) led to a high yield of AH compounds, while a high catalyst-to-feedstock composition (5:1) boosted phenol demethoxylation and alkylation. A cooking oil waste-to-lignin residue composition of 1:1 produced the maximum synergistic effect (52%) and the maximum mono-AH selectivity (83%). Among the compounds converted to AHs by co-pyrolysing waste cooking oil and lignin monomers, guaiacol was the most active, followed by o-cresol and phenol.

Huang et al. (2022) investigated the thermal behaviors, product compositions, and reaction mechanisms of *Chlorella vulgaris* and urea using an HZSM-5 catalyst [47]. The TGA outcomes showed that the introduction of urea reduced the peak degradation temperature of *C. vulgaris* to 342 °C. Further, the urea addition reduced the activation energy values of the mixture. The estimated activation energy values of the blends (113-132 kJ/mol) were smaller than that of the pyrolysis of *C. vulgaris* (208 kJ/mol). The experimental results demonstrated that co-pyrolysis increased the formation of oxygenates. Nonetheless, it reduced the long-chain nitrile content in nitrogen compounds and ester composition in oxygenates. The addition of the HZSM-5 catalyst improved the deoxygenation reaction while inhibiting NH_3_ release. The synthesis of nitriles, olefins, AHs, and non-hydrocarbon compounds was also improved due to the catalyst’s addition. The research established that there is a synergistic influence between the two feedstocks during co-pyrolysis, and the introduction of HZSM-5 promoted urea and *C. vulgaris* co-pyrolysis.

Kim et al. (2017) used TGA and Py-GC/MS analysers to conduct epoxy-printed circuit board (e-PCB) and PP/HDPE co-pyrolysis studies with one type of HZSM-5 (SiO_2_/Al_2_O_3_ 80) catalyst and two types of HY (SiO_2_/Al_2_O_3_ 80 and SiO_2_/Al_2_O_3_ 30) catalysts [48]. Without catalysts, the co-pyrolysis of feedstocks produced no apparent debromination effect. The pyrolysis decomposition of e-PCB using HY(80) and HZSM-5(80) catalysts exhibited a debromination effect. However, it generated only small quantities of bisphenol and brominated phenols. The addition of HDPE/PP to e-PCB pyrolysis over both HZSM-5 and HY catalysts displayed varying debromination efficiencies. HY(80) actually outperformed HZSM-5(80). Because of the large pore size of HY(80), e-PCB co-pyrolysis employing the HY(80) catalyst was highly effective in removing large-sized brominated bisphenol. The catalytic co-pyrolysis process, which used both HZSM-5 and HY catalysts, produced large quantities of useful products, including BTEXs and phenol, which can be commercialised.

Hou et al. (2022) used TG-FTIR and Py-GC/MS analysers to investigate the co-pyrolysis behavior of oil sludge with rice husk waste under the influence of the ZSM-5 catalyst [49]. According to the TGA, the first stage of co-pyrolysis degradation was primarily by rice husk, while the second stage was majorly by oil sludge. At 150 °C, the supplement of ZSM-5 catalyst encouraged the degradation of the feedstock mixture but had only a minor effect on their weight reduction at high temperatures. Rice husk pyrolysis produced aldehydes (CHO), ketones (C=O), and CO_2_ at small temperatures (<350 °C), wherein oil sludge pyrolysis produced aliphatic hydrocarbons at high temperatures (around 450 °C). Under ZSM-5’s influence, oxygenated compounds from rice husk pyrolysis and aliphatics derived from oil sludge pyrolysis were converted into AH compounds. Pyrolysis experiments showed that the co-pyrolysis of oil sludge and rice husk with no catalyst addition had only a minor effect on product composition, whereas catalytic co-pyrolysis had a significant effect. A higher AH yield of 27% was detected for the catalytic co-pyrolysis of feedstocks (mass ratio of 1:1) compared to individual pyrolysis of oil sludge (6% AH yield) or rice husk (8% AH yield). The major AH components that were generated during the co-pyrolysis were benzene, toluene, and xylene (BTX), which had the highest selectivity of 60%, indicating that the co-pyrolysis oil has a high potential as a chemical feedstock.

TGA and tandem micro-reactor (TMR)-GC/MS analysers were utilised by Park et al. (2021) to examine the catalytic co-pyrolytic degradation characteristics of Kukersite oil shale and black pine wood employing zeolite catalysts such as HBeta(25), HZSM-5(Si/Al_2_O_3_:23), and HY(30) [50]. Due to differences in the composition of zeolite types, TGA results showed different decomposition temperature zones for pine wood and oil shale samples. Surprisingly, even after co-feed and catalyst additions, the peak degradation temperature of pine waste did not vary. According to the TMR-GC/MS results, pine waste pyrolysis produced oxygenates without a catalyst, whereas oil shale produced light hydrocarbons. However, the synthesised lightweight hydrocarbons and oxygenates were introduced to AHs by catalysts. HZSM-5(23) exhibited the maximum efficiency for AH synthesis during co-pyrolysis, followed by HBeta(25) and HY(30). This is because the catalysts differ in terms of their pore properties and acidities.

**Figure 4:**
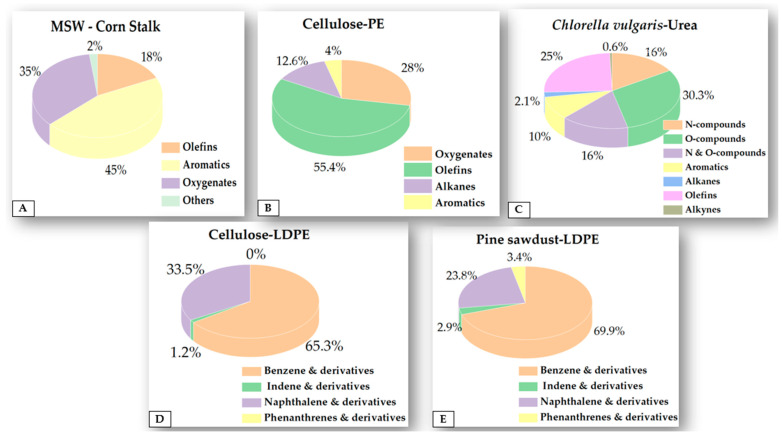
Comparison of articles on HZM: (**A**) MSW and cornstalk [25]; (**B**) CE and PE [35]; (**C**) *Chlorella* [28]; (**D**,**E**) CE and pine dust with LDPE [34].

Using an HZSM-5 catalyst, Wang et al. (2017) explored the fast catalytic pyrolysis of used cooking oil and used tea leaves [51]. Investigations were done into how the H/C ratio and pyrolysis temperature affected the product compositions and AH selectivity. Increases in temperature have been shown to increase AHs from 5.88% to 43.26% in the case of waste dahlia flowers due to increased decarboxylation reactions [52]. The experimental findings by Wang et al. showed that the H/C ratio significantly affected the carbon yields of AHs and olefins. The synergistic effect was improved, and the yields of olefins and AH were significantly increased with an increase in the H/C ratio. The outcomes of the experiments demonstrated that the pyrolysis temperature significantly influenced the compositions of condensable organic products. The yields of olefins and carbon in AHs initially increased as temperatures rose, but a subsequent rise after an optimum temperature resulted in a reversing trend. The optimal temperature for obtaining the highest AHs and olefin yields was determined to be 600 °C. In order to establish the effect of the feeds over HZM on compound generation, Figure 4 depicts some of the studies discussed in the above sections [25,28,34,35]. Table 1 shows the methodologies and key findings of zeolite-based catalysts in some articles reviewed in this paper.

**Table 1:** Findings of zeolite catalysts from selected articles reviewed in this study.

Feedstock	Catalysts	Pyrolysis Operating Conditions	GC-MS Operating Conditions	Products	Reaction Mechanism	Reference
Names: Mixing/blending ratio: BR	Name: Catalyst to feed ratio/loading: CR Catalyst to catalyst ratio: CCR	Instrument (I): Temperature (T): °C Heating rate (HR): Time (t): Carrier gas and flow rate (CG): mL/min	Instrument (I): Capillary column (CC): Temperature: (T): °C Split ratio (SR): Scan range (SC): m/z	→: yields ↓: decreases ↑: increases		
MSW (PP, PE and humus) –corn stalks BR: 3:1. 1:1, 1:3, 3:1, 1:1, 1:3	HZSM-5, nMFI	I: CDS5200 (CDS Analytical Co. Ltd., Oxford, PA, USA) T: 600 °C HR: 20 °C/ms t: 20 s CG: He	I: Agilent 7890B-5977A CC: HP-5 ms T: 250 °C SR: 1:50 SC: 33–500 m/z	➢ Without catalysts, co-pyrolysis → oxygenated organics ↓, olefins ↑ ➢ With catalysts → ↑ AHs, ↑ small-chained olefins	Under ZSM-5, cyclization and aromatization of olefins turn feedstock into AHs. Diels–Alder reactions of C6-C10 olefins with furans were more frequently observed with nMFI with more Lewis acid sites than with ZSM-5.	[25]
*Chlorella vulgaris*–MSW BR: 1:1	ZSM-5, Hi-ZSM-5, Al-MCM-41, Al-SBA-15 CR: 1:1	I: CDS5200 (CDS Analytical Co. Ltd., Oxford, PA, USA) T: 600 °C HR: 10 °C/min t: 20 s CG: N_2_	I: Agilent 7890B-5977A CC: HP-5 ms T: 250 °C SR: 1:50 SC: 35–300 m/z	➢ ↑ monocyclic AH selectivity (34.14%) ➢ ↓ acid selectivity (9.54%)	Co-pyrolysis increased mono-AHs and aliphatic hydrocarbons while decreasing poly-AHs and N_2_-compounds.	[28]
Chili straw–PP BR: 1:1	HZSM-5 CR: 1:1	I: Pyroprobe 5200 Pyrolyser (CDS Analytical Co. Ltd.) T: 750 °C HR: 1.2 × 10^6^ °C/min t: 10 min CG: N_2_	I: Agilent 7890B-5977A CC: HP-5 ms T: 300 °C	➢ HZSM-5 → ↑AHs from 4.46% to 17.34%, ↓ from 12.75% to 7.71% ➢ Microwave power 568 W and HZSM- 5→ oxygenated compounds ↓ from 17.41% to 13.09%	The alkane content increased as the micro power level increased, but the oxygenate organics decreased.	[29]
CE or pine sawdust–LDPE BR: 1:1	HZSM-5 CR: 1:1	I: A specially designed fixed-bed reactor T: 500 °C t: 30 min CG: N_2_ at 150 mL/min	I: ITQ 900 Instrument CC: HP-5 ms T: 280 °C SR: 1:10 SC: 30–500 m/z	➢ ↑ → reaction activity and reduced activation energy ➢ Biomass and LDPE → ↑ selectivity for AHs, BTEX. ➢ LDPE → ↑ methylnaphthalene and 2-methylnaphthalene ➢ HZSM05 → ↓ AHs larger than C10	The yield of AHs is increased by Diels–Alder reactions between oxygenated compounds derived from biomass and olefins derived from plastic.	[34]
CE–PE BR: 1:1	HZSM-5 CR: 1:4	I: EGA/PY-3030D pyrolyser T: 650 °C HR: 20 °C/ms t: 30 s	I: QP2010Ultra (Shimadzu, Japan) CC: DB-5 ms T: 280 °C SR: 1:80 SC: 33–500 m/z	➢ ↑ AHs and olefins ➢ ↓ oxygenated compounds	The addition of HZSM-5 advances the Diels–Alder reaction, suppressing the free radical reactions. The amount of the alcohol compound is decreased in this.	[35]
Quercus Variabilis–waste plastic film BR: 1:1	HZSM-5 or HY CR: 1:5	I: Pyrolyser (Py-3030D, Frontier Laboratories Ltd., Fukushima, Japan) T: 600 °C	I: Agilent 7890A/5975C CC: UA-5 T: 600 °C SR: 1:100 SC: 15–800 m/z	➢ ↑ AHs and ↓ coke ➢ ↑ benzene, toluene, ethylbenzenes, xylenes, poly-AHs, C2-C4 olefins for HZSM-5 ➢ C1-C4 alkanes higher for H5 1:5	The catalytic cracking effects of the mixture produced significantly more light olefins (C2~C4) and then converted them to AHs via a Diels–Alder reaction.	[36]
Poplar wood sawdust–HDPE powder BR: 1:1	ZSM-5 (0.1, 0.2, 0.3, 0.5, 0.7 M sulphuric acid) CR: 1:1	I: CDS Pyro Probe 5200 HP Pyrolyser (Chemical Data Systems) T: 500 °C	I: Agilent 7890B (GC) Agilent 5977B (MS) CC: HP-5ms T: 300 °C SR: 1:50	➢ Co-pyrolysis → ↑ olefin (53.32%) ➢ ↓ oxygenates except alcohol. ➢ Acid-modified ZSM-5 → olefins between 56.20% and 59.7% ➢ Alkane → 23.29-25.96% ➢ ZSM-5 (0.5 M) → highest AHs (12.72%) and lowest HC without acid treatment (76.84%)	Several processes contribute to the formation of AHs at the Bronsted acid site, including dehydrogenation cracking, oligomerization, Diels–Alder reaction, cyclization dehydrogenation, and the hydrocarbon pool mechanism.	[43]
Poplar wood–PP BR: 1:1	HZSM-5, HBeta, HY, HUSY CR: 1:4	I: Pyrolyser (CDS5200HP-R) T: 600 °C HR: 20 °C/ms t: 60 s	I: Agilent 6890 (GC) and mass detector Agilent 5973 CC: DB-17 ms T: 280 °C SR: 1/50 SC: 40–500 m/z	➢ AHs ↑ → HZSM-5 > HY > HBeta > HUSY ➢ Coke yield lowest for HZSM-5 (6.4%), ↑ ➢ HZSM-5 → ↑ poly-AHs ➢ HBeta → ↑	To varying degrees, all synergies increased alkene yield. HBeta demonstrated greater synergistic deoxygenation than HZSM-5. Furthermore, the synergistic effects of HBeta and HY promoted the formation of mono AHs, whereas HZSM-5 increased the selectivity of poly-AHs.	[38]
CE–PP BR: 1:1	MCM-41 and Al-MCM-41 CR: 1:10	I: 5200HP, CDS, USA T: 300 °C, 400 °C, 500 °C, 650 °C HR: 20 °C/ms t: 18 s CG: He at 1	I: Clarus 560S, PerkinElmer, Shelton, CT, USA CC: DB-5MS T: First: hold 50 °C for 4 min; second: heat to 280 °C at heating rate of 3 °C/min; final: hold for 20 min at 250 °C SR: 1:20	➢ Co-pyrolysis → ↓ yields of furans ➢ → ↑ yields of olefins and AHs with ↑ in P ➢ → ↑ of furans with ↑ by catalyst (MSM-41) and ↑ olefins and AHs by Al-MCM-41	Catalytic co-pyrolysis using Al-MCM-41 involves acid centers within zeolites, hydrocarbon reactions, and Diels–Alder reaction	[39]
CE–PP BR: 1:1	(Ni)-MCM-41 CR: 1:10	I: 5000 HP, CDS, Oxford, PA, USA T: 650 °C HR: 20 °C/ms t: 20 s CG: He at 1	I: Thermo Scientific, Trace DSQII) T: First: hold at 50 °C for 4 min; second: heat to 280 °C at a rate of 3 °C/min; final: maintain at 280 °C for 12 min SR: 1:20	➢ Ni ↑ synergy → increased acidity of catalyst. ➢ Best catalytic performance for a Ni loading of 25.1 wt.% (Cat-C)	Ni → active metal sites → ↑ bond breaking ability and deoxidation performance of catalysts → promotes reaction pathways → olefins and AHs	[40]
Laminaria japonica–PP BR: 1:1	Zeolite catalyst CRl 1:1	I: Frontier-Lab Co., Py-2020iD, Fukushima, Japan T: 500 °C	I: Agilent Technology, 7890A/5975i, Santa Clara, CA, USA CC: UA-5 capillary column	➢ Catalytic co-pyrolysis → bio-oil content of ↓ oxygenates. ➢ → ↑ AHs, wax to lighter hydrocarbons	Acid sites on catalyst → ↑ AHs while the pore size of the catalysts was more important in removing oxygenates and wax species.	[41]
Water hyacinth–scrap tire BR: 1:1	HZSM-5 and multilamellar MFI nanosheets. CR: 2:1, 1:1, 1:2 and 1:4	I: Py: CDS5200, CDS Analytical Co. Ltd. T: 600 °C HR: 20 °C/ms t: 20 s CG: He at 1.	I: GC/MS: Agilent 7890B-5977A)	➢ Co-pyrolysis → significant synergistic effects ➢ Multilamellar MFI nanosheets HZM-5 → ↑ removal of oxygenates and conversion of aliphatic hydrocarbons into mono-AHs	Multilamellar MFI → multiple pore types and high accessibility of acidic sites → access mono-AHs and BTEXs.	[42]
*Chlorella vulgaris*–urea BR: 2:1, 1:1, 1:2	HZSM-5 CR: 1:0.25	I: Pyrolyser (CDS5200, CDS Analytical Co. Ltd.) T: 600 °C HR: 20 °C/ms t: 60 s	Agilent 7890B-5977A CC; HP-5 ms T: 280 °C SR: 1/60	➢ Co-pyrolysis → ↑ oxygen-containing functional groups HZSM-5 ➢ → ↑ deoxygenation reactions, ↓ NH_3_	The addition of HZSM-5 accelerated the removal of oxygen-containing groups in acids and esters as well as the cleavage of their long chains, converting acids and esters to olefins.	[47]
Biomass carbohydrate–LLDPE BR: 1:1	Mesoporous ZSM-5 (MZSM-5) and Al-SBA-15 CR: 2% of catalyst	I: Tandem μ-reactor (RX-3050TR, Frontier Laboratories, Fukushima, Japan) T: 500 °C	I: GC/MS (7890A/5975C inert, Agilent Technology, Santa Clara, CA, USA)	➢ MZSM-5 → synergistic effect on AHs ➢ → Higher yield of mono-AHs	Catalysts produced micropores and mesopores, causing larger mono-AHs due to shape selectivity to form AHs and diffusion of large molecular pyrolysates.	[37]

## 3. Zeolites in Combination

### 3.1. Biomass-Biomass with Combined Zeolites

A unique publication [53] employed an orthogonal experimental design to understand the influence of operating temperatures, catalyst type, and mixing ratio of two biomass wastes (*camellia* shell and take-out solid waste). The targeted reduction of acids and increase in monocyclic AHs were possible due to a combined effect of HZSM-5 and CaO, high take-out solid wastes (3:7), and high temperatures (700 °C). While the CaO increased the ketones content due to neutralisation reactions, deoxygenation reactions at the acid sites of HZM-5 increased AHs. A combination of metal oxides (NiO + MoO_3_) and ZSM-5 also increased the AHs during the co-pyrolysis of sewage sludge and sawdust. In contrast, the metal oxides promoted furan, ketone, and phenol formations, and the zeolite increased the AHs (by 84.2%) [54]. The poly-AH yields were reduced in a dual catalyst scenario while the phenols and AHs increased. The dual catalyst also hindered aldehyde production, an essential aspect of bio-oil aging reactions and instability.

### 3.2. Biomass-Plastics with Combined Zeolites

More studies investigated the combined effects of zeolites and other catalysts during biomass plastic co-pyrolysis. Comparing non-catalytic and catalytic co-pyrolysis found that the relative content of hydrocarbons (aliphatic and 1-ethyl-3-methyl-benzene) was the highest (71%) when the waste greenhouse plastic films to rice husk ratio was 1.5:1 [55]. A hierarchical molecular sieve catalyst based on HZSM-5/MCM-41 at 600 °C increased the mono-AHs. The catalysts MCM-41 aided in cracking large, oxygenated chemicals first, followed by the shape-selective effects of the HZSM-5 catalyst to form AHs.

Metal oxide-based dual catalysts are gaining attention due to their effectiveness in reducing acids. When CaO and HZSM-5 were combined during the fast co-pyrolysis of hemicellulose and LLDPE, ketone synthesis increased, while acids were reduced to 43 and 3%, respectively, at a catalyst-to-feedstock ratio of 1:2 [56]. Additionally, the hydrogen-rich portions from LLDPE combined with furan and non-furan compounds by undergoing Diels–Alder reactions and hydrocarbon pool reactions to increase hydrocarbon yields significantly. The acid removal was suggested to have occurred via three reaction pathways occurring at various temperatures and in the presence of CaO, including neutralisation, thermal cracking, and catalytic cracking reactions yielding ketones and hydrocarbons. Additionally, CaO prefers producing furans rather than aldehydes. Alternatively, the unique pore structure of HZSM-5 is known to contribute to deoxygenation reactions and, thereby, aromatisation aids in the conversion of ketones and aldehydes to AHs. Considering the catalytic behavior of individual CaO and HZSM-5, the dual catalysts’ behaviour increased AHs (even higher than in the presence of single HZSM-5) and reduced ketones (opposing the effects of single CaO). The deacidification effects were reduced in the presence of the dual catalyst due to decreasing ketones (due to CaO) and further conversion of ketones to AHs (due to HZSM-5).

Two other studies focused on synthesising CeO_2_/γ-Al_2_O_3_ and tandem bed CeO_2_ with HZSM-5 for biomass-plastic co-pyrolysis [57,58]. The former study concluded the role of increasing LLDPE for AH production in the presence of the catalyst, with maximum yields at 75% LLDPE with bamboo sawdust. The increase was seen in alkylbenzenes, ethylbenzenes, and xylenes. Additionally, an optimal mass ratio of 3:1 of HZSM:5 to CeO_2_/γ-Al_2_O_3_ increased AHs significantly, especially pure HZSM-5. The latter study investigated the co-pyrolysis of corn stover and LDPE and found increasing the plastic content aided in the increased yield of aliphatics and BTX due to the Diels–Alder reaction, hydrocarbon pool, and hydrogen transfer reactions, eliminating oxygenates completely, especially in addition to the catalysts. The CeO_2_ in the tandem bed removed oxygens from acids, aldehydes, and phenols, and the HZSM-5 transformed the deoxygenated compounds into AHs and aliphatics.

In contrast, combining sodium carbonate/gamma-alumina and HZSM-5 for biomass (sugarcane bagasse pith) and PET helped to eliminate 18% of the oxygen content, increasing AHs and olefinic compounds more than in the presence of HZSM-5 alone [59]. Furthermore, adding sodium reduced coke formation, which was detrimental to the efficiency of zeolite catalysts. Additionally, an optimal condition of 700 °C at a biomass-plastic ratio of 4 and a catalyst ratio of 5 was suggested for optimal yields of carbon, AHs, BTEX, olefins, and phenols. Adding polyvinyl chloride (PVC) to agro-industrial (almond shell and olive pomace) biomass enhanced BTX yields up to 27% [60]. Furthermore, the mineral content in the catalyst NaZSM-5 enhanced mono-AHs and reduced poly-AHs and oxygen-containing compounds. The alkali and alkaline earth metals are known to have catalytic biomass pyrolysis due to depolymerisation and cracking anhydrosugars; in this case, the total hydrocarbons reached 28% when NaZSM-5 was added to olive pomace due to the inherent presence of alkali and alkaline earth metals (AAEMs) in the feed and the sodium in the catalysts.

### 3.3. Other Feeds with Combined Zeolites

**Figure 5:**
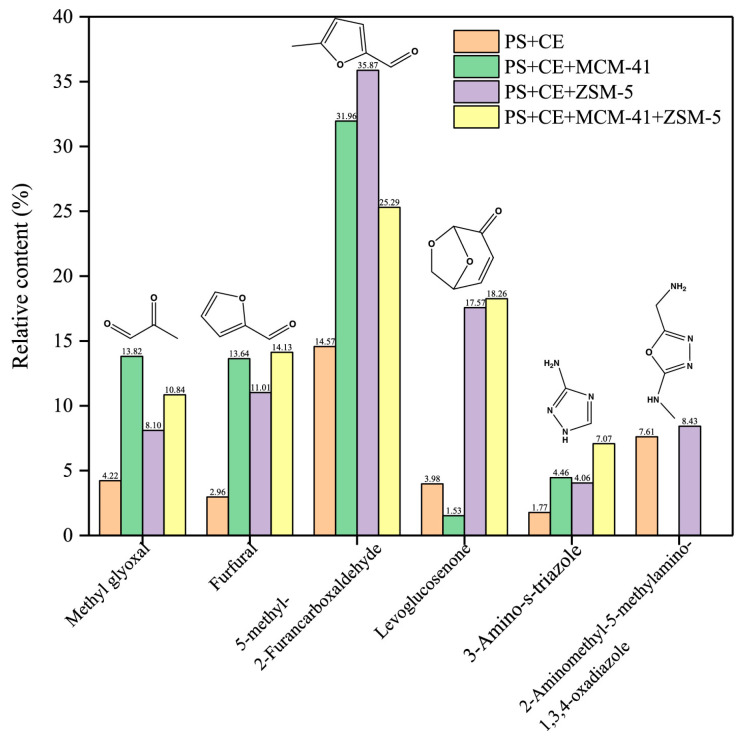
Comparing non-catalytic soluble polysaccharides and CE with different zeolite catalysts MC-41, ZSM-5 and their mixtures [62]. Copyright, 2022, Wiley & sons.

The literature reports the co-pyrolysis of biomass with feeds other than biomasses and plastics. Individual and blended catalysts of MCM-41 and ZSM-5 were studied for the co-pyrolysis of lignocellulosic biomass for enhanced biofuels and high-value chemicals [61]. Interesting observations included the great bio-oil upgrading capability of MCM-41 rather than ZSM-05 and the blends rather than either. The main advantages were owed to the selectivity of the monocyclic AHs due to the transformation of furans via Diels–Alder reactions. The same catalysts induced and reduced the generation of furans and anhydrosugars, respectively, during the catalytic co-pyrolysis of polysaccharides and CE [62]. The furans are known to be used for food and fuel additives, and in the presence of MCM-41 and ZSM-5, the promotion of 2-furaldehyde up to 13.64, 31.96%, and 5-methyl-2-furaldehyde can reach values of up to 11%, and 36%, respectively. However, the content of levoglucosane was much higher in the presence of the latter catalyst. Blending the catalysts with the feeds mainly produced furans (highest with 46%) and levoglucosenone (with active optical compounds) achieved the highest values. The catalysts, therefore, enhanced the furan compounds and reduced anhydrosugars as compared to the non-catalytic pyrolysis. Figure 5 compares the non-catalytic co-pyrolysis with the different catalytic co-pyrolysis.

Two studies focused on fast catalytic pyrolysis in the presence of HZSM-5 in combination with CaO. *Spirulina* (low-lipid microalgae) with oil shale showed the latter delayed thermal degradation of organic matter in the algae [63]. Additionally, the catalyst increased the deoxygenation and aromatization reactions while reducing acids and phenols. Maximum values for AHs, mainly benzene, toluene, and p-xylene, were obtained at a 1:3 ratio of CaO and HZSM-5L; the increase was 2% higher than individual HZSM-5. Additionally, the significant decrease in poly-AHs—by 7%—with increasing CaO proportions helped reduce coke formation and, thereby, the deactivation of zeolites. Similar effects were observed when bamboo residual and waste tires were co-pyrolysed using the same catalysts [64]. There was a decrease in acid formations and an increase in AH and olefin production, with maximum hydrocarbon yields achieved at a mass ratio of 3:2 (zeolites:HZM:5) [64]. The future perspective in their paper suggested the utilisation of benzene in the production of polymers, toluene, and p-xylene for gasoline fuels, as well as in the production of polymers such as polyurethanes and PET. Another study analysed the co-pyrolysis of bamboo residue with waste lubricating oil, while MgO reduced acids and increased ketone yields by aldol condensation reactions and ketonisation [65]. Waste lubricating oil increased yields of olefins, alkanes, and AHs specifically due to the hydrocarbon pool mechanism and played the role of a hydrogen donor, therefore being a useful additive in bio-oil additives. Mixing the catalysts MgO and HZSM-5 in the ratio of 2:3 further accelerated the yields of AHs via Diels–Alder reactions. Table 2 shows the methodologies and key findings of zeolite-based catalysts in combination with other catalysts from some articles reviewed in this paper.

## 4. Catalysts Other Than Zeolites

This section of the paper discusses the limited literature available on using catalysts other than zeolite for pyro-probe pyrolysis. Generally, some metals such as nickel [66], copper [67], iron [68], and AAEMs, such as sodium, potassium, magnesium, and calcium, are employed as catalysts in pyrolysis processes [69]. Additionally, metal oxides such as aluminum, zinc, and iron oxide are considered important catalysts [70]. Their low cost, accessibility, and effectiveness in removing oxygen make them suitable catalysts. Carbon-based catalysts such as activated carbon, carbon black, and biochar are being increasingly researched [71,72,73]. Furthermore, acid and base catalysts are also often considered for pyrolysis processes [74,75]. All these catalysts are known to enhance the yield of bio-oil and syngas and improve the quality of the products. The choice of catalyst is often made depending on the type of catalyst and the desirability of the products.

Some studies in this section deal with self-catalysis, the catalysis that occurs when the feed components possess catalytic properties, improving the quality and quantity of the products [76]. An example of such a catalyst is red mud, a by-product of the Bayer process of extracting aluminium from bauxite ore. Red mud contains a mixture of iron oxides, titanium oxides, and other minerals [77]. The main attraction is the high content of iron oxide, enabling larger hydrocarbon breakdowns to take place. This article discusses one such study that utilised beechwood with red mud catalyst in various ratios (Table 1). This study shows how increased red mud ratios improve product yields such as acid, furfural, aldehyde, and ethylenediamine [78]. Additionally, the synthesis of phenols, CO_2_, esters, alcohols, and a few others was reduced. The results suggest product generation is not linearly related to the catalyst loading, as complex, competing reactions mechanisms, including the rupture of f C_β_-C_γ_ bonds leading to alkyl chain changes in lignin, secondary decomposition of anhydrosugars, a CE decomposition product, ring scission, and rearrangements leads to such a result [78]. Especially for the reduced CO_2_ generation, the water gas shift reaction and competitive decarboxylation/decarboxylation at catalyst sites played a key role.

**Figure 6:**
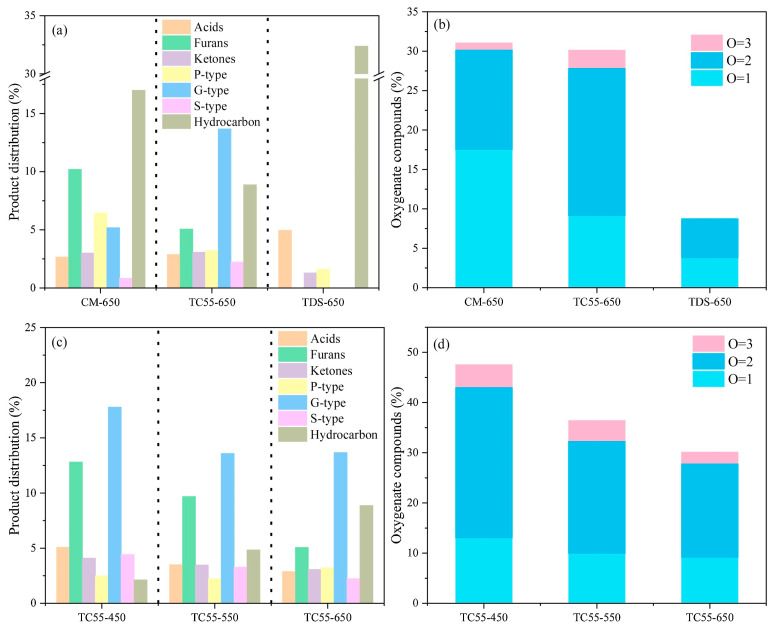
Cattle manure (CM), Textile dyeing sludge (TDS) and co-pyrolysis in equal proportion (TS55) product distribution at 650 °C (**a**) at varying temperatures (**c**), oxygenated compounds at 650 °C (**b**), and varying temperatures (**d**) [79]. Copyright 2022, Elsevier.

Another study on textile sludge with cattle manure pyrolysis suggested that the presence of iron in the sludge samples improved the interaction of the feedstocks, catalysed the reaction, and advanced the release of volatiles in the early pyrolysis stage [79]. The co-pyrolysis of the blend also led to the change in mechanism from a diffusion model to a reaction-order model. Figure 6 shows how the relative yield of non-aromatic oxygenates, such as acids, ketones, and furans, reduced with an increase in temperature. These compounds changed into alkanes, increasing the hydrocarbon content. Furthermore, co-pyrolysis also improved the G-type compounds in the volatiles.

Another study on microalgae and oil shale co-pyrolysis used oil shale, an organic, rich, carbonaceous sedimentary rock known to process 90% of kerogen to raw materials of fuels and valuable chemicals [80]. The presence of oxides, namely SiO_2_ and Al_2_O_3_, and the presence of metals such as copper, chromium and zinc make the oil shale catalytic [80]. The presence of oil shale during the fast pyrolysis maximised the AH yields to 35%, with only 3% of OS enabled by catalytic cracking and reforming. Additionally, the high H/C ratio of OS enhanced the production of hydrocarbons and olefins by promoting the Diels–Alder reaction towards cycloaddition [80]. A similar study used microalgae, *Chlorella vulgaris*, and kitchen wastes in different ratios, with different catalysts, including CaCO_3_, CaO, SiO_2_, and permutit [81]. Among the catalysts, CaO positively affected pyrolysis by reducing the acid content drastically and improving the hydrocarbon and BTEX contents. The requirement for future mechanism-related studies was suggested elsewhere [81].

CE is another feed considered to cause synergistic effects with many feeds, thereby resulting in improved production of useful products such as anhydrosugars, furans, alkylphenols and olefins [82]. The following two studies utilised CE for pyrolysis with strong-acid cation exchange resin [83] and HDPE [82]. Both these studies provide profound results using Amberlyte-15 catalyst and alkali earth metals, respectively. The former study concluded a temperature range below 400 °C led to increased production of anhydrosugar, levoglucosenone, and 1,6-anhydro-β-d-gluco-furanose from CE- and inorganic sulphur-containing species from the sulfonic groups of the A15 catalyst; increasing temperature further improved the production of monocyclic and polycyclic AHs, especially organic sulphur-containing compounds [83]. The second study utilising alkali earth metals as catalysts co-pyrolysed CE and HDPE; sodium showed the strongest catalytic cracking, followed by potassium, calcium, and magnesium. Similar to the previous work, the synergistic effects facilitated the formation of anhydrosugars due to the cleavage of glycosidic bonds and the breakage of HDPE to generate hydrocarbons [82]. Regarding other products such as furans and ketones, potassium served as a better catalyst, as it promoted ring-opening reactions and reduced glycosidic bonds, inhibiting the synergy between the feeds [82].

As shown in this section of the paper, there are only a few studies that have used catalysts other than zeolites for pyrolysis. As a recommendation, future studies should focus on feeds with lignin and CE due to the catalytic behaviour breaking down large molecules into small ones. Similarly, clay materials with catalytic behaviour also need to be studied [84]. Sometimes, the availability of certain aforementioned catalysts is limited by the availability, cost, and environmental impact. Therefore, studies on the use of low-cost catalysts, especially from waste sources, should be conducted. Table 3 shows the methodologies and key findings other than zeolites of some articles reviewed in this paper.

## 5. Summary and Future Work

Studies focus on the effect of adding catalysts on the volatiles generated from co-pyrolysis using a micro-pyrolyser with GC-MS. Adding zeolites increased aromatization and short-chain olefins and reduced residual weights. Using nMFI aided in increased furans due to Diels–Alder reactions. A comparative study found that H-ZSM-5-40 is better than other zeolites due to its steric hindrance and low pH. Additionally, another study found the presence of both micropores and mesopores of MZSM-5 produced more mono- AHs compared to the presence of Al-SBA-15. The addition of the HZSM-5 catalyst inhibited NH3 release and improved the synthesis of nitriles, olefins, AHs, and non-hydrocarbon compounds. The catalytic co-pyrolysis process, which used both HZSM-5 and HY catalysts, produced enormous quantities of useful products, including BTEXs and phenol, which can be commercialized. Furthermore, the mineral content in the catalyst NaZSM-5 enhanced mono-AHs and reduced poly-AHs and oxygen-containing compounds. Additionally, acid-modifying ZSM-5 produced a higher olefin yield (53%) than individual poplar pyrolysis (17%) due to increased acid sites for catalytic activity.

The feed also poses a significant effect on the products; for example, mixing cellulose and plastics increases AHs due to Diels–Alder reactions between olefins and furans. A unique study showed the highest value of 71% of BTEX at optimised conditions of 650 °C, 0.5 MPa pressure, a 1:1 PE/lignin ratio, and a 4:1 catalyst/raw material ratio. On the other hand, biomass–LDPE co-pyrolysis increased AH yield and selectivity while decreasing selectivity for BTEX due to synergistic effects, therefore reflecting the importance of the biomass type. Additionally, adding a high percentage of cooking oil in the feedstock mixture and an ideal catalyst (HZSM-5)-to-feedstock ratio (3:1) led to a high yield of AH compounds, while a high catalyst-to-feedstock composition (5:1) boosted phenol demethoxylation and alkylation. Another study showed that waste lubricating oil increased olefin, alkane, and AH yields specifically due to the hydrocarbon pool mechanism and played the role of hydrogen donors.

Combining zeolites found significant product yields due to individual catalytic activity. For example, while CaO increased the ketone content due to neutralization reactions, deoxygenation reactions at the acid sites of HZM-5 increased AHs. Additionally, the poly-AH yields were reduced in a dual catalyst scenario, while the phenols and AHs increased based on HZSM-5/MCM-41 at 600 °C. The CeO_2_ in the tandem bed removed oxygens from acids, aldehydes, and phenols, and the HZSM-5 transformed the deoxygenated compounds into AHs and aliphatics corn stover and LDPE. Blending MCM-41 and ZSM-5 with the feeds mainly produced furans (with the highest value of 46.24%), and levoglucosenone (with active optical compounds) was the highest.

This paper’s final section discusses the limited literature available on using catalysts other than zeolite for fast pyrolysis studies. Catalysts for pyrolysis processes include metal oxides, carbon-based catalysts, acid, and base catalysts, as well as metals such as nickel, copper, iron, and AAEMs. The choice of catalyst is often made depending on the type of catalyst and the desirability of the products. This review also discussed feedstocks that behave as catalysts themselves, known as self-catalysis, and that utilise components present in the feed that possess catalytic properties to improve the quality and quantity of the products. An example of such a catalyst is red mud, a by-product of the Bayer process that contains a mixture of iron oxides, titanium oxides, and other minerals. Another example is oil shale, an organic-rich, carbonaceous sedimentary rock known to process 90% of kerogen to raw materials of fuels and valuable chemicals. The presence of oxides and metals in oil shale makes it catalytic, and it maximises AHs yields to 35%, with only 3% of OS enabled by catalytic cracking and reforming. Additionally, CE is another feed that causes synergistic effects with many feeds, resulting in improved production of useful products, such as anhydrosugars, furans, alkylphenols and olefins.

This review has shown the significant research that is being conducted in the field. However, the drawbacks and limitations of the technology could guide future work. Py/GC-MS, despite its many advantages, also has several disadvantages that should be considered [10]. Py/GC-MS has several disadvantages, including its destructive nature, which alters or destroys the sample during analysis, and the difficulty in further performing qualitative analysis of a bio-oil. Additionally, complex mixtures can produce a vast number of pyrolysis products and fragments, making it challenging to interpret the resulting mass spectrum. Reproducibility can also be problematic in interlaboratory comparisons, as different materials and methods of pyrolysis and GC analysis can influence the results. The strict standardisation of all experimental conditions is necessary to achieve reliable and accurate results.

Future work should focus on determining the rate constants for various feedstocks and catalysts while analysing the by-products of the co-pyrolysis using analytical methods, such as Fourier-transform infrared spectroscopy (FTIR) and nuclear magnetic resonance (NMR) spectroscopy. Studies should investigate the stability, long-term usability, and prospective applications of the catalysts and the catalytic co-pyrolysis products. Additionally, targeted products are more useful for application; therefore, studies should focus on a reporting standard for the products. Although the volatiles increase with increasing heating rate and significantly reduce coke formation [85], such studies for co-pyrolysis using py/GC-MS need to be conducted. Furthermore, research carrying out large-scale experiments (to confirm the findings of microscale research) to determine whether it is feasible to include catalytic co-pyrolysis into industrial processes for environmentally friendly waste management and energy generation is necessary.

## 6. Conclusions

The increasing interest in sustainable energy resources has led to the exploration of biomass and biofuels as alternative sources to decrease dependence on fossil fuels and mitigate the impacts of climate change. Py-GC/MS analysis, which combines pyrolysis, gas chromatography, and mass spectrometry, has greatly improved researchers’ understanding of the reaction mechanisms and factors influencing the process. This review aimed to provide an updated understanding of the latest developments in catalytic biomass fast co-pyrolysis, including the catalysts, reaction conditions, products, and intermediates formed. Zeolites are aluminosilicate minerals with a unique pore structure and acidity, making them ideal catalysts for deoxygenation and cracking reactions. Several studies have explored the use of zeolite catalysts in the co-pyrolysis of various biomass types, including municipal solid waste, corn stalks, rice husks, cedar, marine eelgrass, and *Chlorella vulgaris*. The results show that using zeolite catalysts improves the hydrocarbon content of the pyrolysis products and enhances the conversion efficiency of raw materials to bio-oil. Different zeolites, such as ZSM-5 and nMFI, have varying effects on the composition of the pyrolysis products. Moreover, the co-pyrolysis of biomass and plastics using zeolite catalysts such as HZSM-5 increased the yield of hydrocarbons and AHs. The paper also discusses the limited literature available on catalysts other than zeolites and their impact on fast pyrolysis processes. Metal oxides, carbon-based catalysts, acid and base catalysts, and self-catalysts (such as red mud, oil shale, and CE) improve the quantity and quality of pyrolysis products. Future work is discussed at the end of the paper, outlining the need for upscaling technologies, and understanding the stability of both catalysts and products.

## Figures and Tables

**Table 2:** Findings of zeolites in combination with other catalysts from selected articles reviewed in this study.

Feedstock	Catalysts	Pyrolysis Operating Conditions	GC-MS Operating Conditions	Products	Reaction Mechanism	Reference
Camellia shell–take-out solid waste BR: 0:1, 0.7:1, 0.5:1, 1:0	HZMS-5, CaO, MgO CR: 1:2 CCR: HZSM-5 with CaO and MgO is 1:1	I: Pyro probe 5200 Pyrolyser (CDS Analytical Co. Ltd.) T: 500–700 °C HR: 20 °C/ms t: 20 s CG: N_2_	I: Agilent 7890B-5977A CC: HP-5 ms, 30 m × 0.25 mm ID 0.25 μm T: 50 to 250 °C HR: 10 °C/min SR: 1:50 SC: 30–500 m/z	➢ ↑ mono-AHs, **↓** acids. ➢ ↑ take-out waste → ↑ aliphatics and ↓ acids	The takeout solid waste process, which contains more hydrogen, enhances the conversion of oxygenated compounds to hydrocarbon products.	[53]
Sewage sludge-–awdust BR: 1:2	MMOs CCR: 1:1 of NiO and MoO_3_ to form MMOs. MMOs modified with ZSM-5 refers to MMOs + ZSM-5 (50%) CR: 1:1	I: CDS pyro probe model 5200 Pyrolyser T: 600 °C HR: 30 °C/min t: 10 s CG: He	I: GC/MS (Shimadzu GC/MS-QP 2010 Ultra) CC: Rtx-5 polar column T: 40 to 270 °C HR: 8 °C/min SR: 1:50 SC: 35 and 500 m/z	➢ Combining catalysts → ↓ light and heavy phenols, AHs ➢ Reduced poly-AHs by 70.5%	The use of both prohibited aldehydes and a specific reaction pathway improves the production of phenolic compounds, both light and heavy. The use of MMOs (manganese oxide) increases the yield of light phenols by promoting demethoxylation, decarbonylation, and cracking reactions.	[54]
Waste greenhouse plastic films– rice husk	HZSM-5/MCM-41 CR: 1:2	I: CDS Pyro probe 5200 Pyrolyser HR: 20,000 °C/s t: 20 s CG: He	I: Agilent Technologies, 7890A/5975C, 2010, Santa Clara, CA, USA CC: DB-5 ms capillary column (0.25 mm × 0.25 μm × 30 m) T: from 50 to 290 °C HR: 8 °C/min.	➢ Co-pyrolysis → ↑ hydrocarbons (max 600 °C) ➢ ↓ acids to almost zero ➢ ↑ hydrocarbons compared to single catalyst (HZSM-5)	HZSM-5 catalyzes deoxygenation reactions by its shape selectivity. When plastic and biomass are used together in co-pyrolysis, oxygenated chemicals are produced, leading to the breakage and fragmentation of long-chain organic molecules. This promotes the production of hydrocarbons, which is the desired outcome for bio-oil production.	[55]
Hemicellulose: LLDPE BR: 0 to 100 wt.%	CR: 1:2 CCR: CaO to HZSM-5—1:0, 2:1, 1:1, 1:2 and 0:1	I: CDS Pyro probe 5200 T: 450–700 °C HR: 20,000 °C/s t: 20 s	I: GC/MS, Agilent 7890A/5975C CC: DB-5 ms capillary column (length = 30 m, I.D. = 0.25 mm, film thickness = 0.25 μm T: 30 to 290 °C HR: 8 °C/min SR: 60:1 SC: 25–550 m/z	➢ ↑ hydrocarbons ➢ ↓ acids	LLDPE enhances hydrocarbons (alkene) due to Diels–Alder reactions and hydrocarbon pool reactions. Increases AHs from∼27% over sole HZSM-5 to utmost ∼40%. CaO deoxygenated the acids, HZM-5 aromatization of ketones.	[56]
Bamboo sawdust– LLDPE	CeO_2_/γ-Al_2_O_3_ and HZSM-5 CCR: HZSM-5 and synthesized CeO_2_/γ-Al_2_O_3_ with 8 wt.% CeO_2_ loading	I: CDS Pyro probe 5200 Pyrolyser T: 600 °C HR: 2000 C/s t: 30 s CG: He	I: GC/MS, 7890A/5975C, Agilent) CC: HP-5MS, 0.25 mm × 0.25 μm × 30 m T: 40 to 180 °C at a heating rate of 5 °C/min, and then was increased to 280 °C at 10 °C/min SC: 28–350	➢ CeO_2_/γ-Al_2_O_3_ to HZSM-5 mass ratio ↑ → AHs ↓ first then ↑	AHs increased at first and then decreased as the LLDPE percentage was elevated from 20% to 100%. Increasing LLDPE (due to the additive effect of monocyclic AHs) favours the production of xylenes, ethylbenzene, and alkylbenzenes. The olefins from LLDPE pyrolysis favors the Diels–Alder cycloaddition and AH production.	[57]
Corn stover: LDPE BR: LDPE to CS was set at 1:5, 1:2, 1:1, 2:1 and 5:1	CeO_2_ and HZSM-5 in tandem CRR: CeO_2_ to HZSM-5—1:5, 1:2, 1:1, 2:1, and 5:1	I: CDS Pyro probe 2000 Pyrolyser T: 600 °C HR: 10,000 °C/s t: 30 s CG: He	I: Agilent 7890A/5975C GC/MS T: 40 to 280 °C at 5 °C/min to a final temperature of 280 °C SR: 60:1 SC: 30–500 m/z	➢ ↑ LDPE → ↑ aliphatics, BTX	Catalysis of CeO_2_; aldehydes and acids are converted to ketones. HZSM-5 promotes the Diels–Alder reactions and cyclization and dehydration reactions to form mono-AHs and alkenes.	[58]
Sugarcane bagasse pith– PET BR: biomass to plastic ratio—0 to 5.	HZSM-5 and sodium carbonate/gamma-alumina served. CCR: HZSM-5 to Na_2_CO_3_—1 to 5	I: Rx-300 TR, Frontier Laboratories, Japan T: 400 to 800 °C CG: He	I: 7890A, Agilent Technologies, Santa Clara, CA, USA T: 45 to 280 °C HR: 10 °C/min SR: 1:50	➢ Co-pyrolysis → ↑ AHs and olefins by 8.7% and 6.9% ➢ Biomass-to-plastic ratio = 4, HZSM-5-to-sodium carbonate/gamma-alumina ratio = 5. → AHs and BTEX ↑ to 18.3% and 17%	Plastics support the hydrocarbon and Diels–Alder pathways, reducing coke formation. Additionally, H_2_O decreased, increasing AH compounds (ethylbenzene, toluene, and xylenes). The presence of sodium prevented coke formation.	[59]
Olive pomace or almond shell–PVC BR: biomass to plastic—1:1.5	NaZSM-5 and HZSM-5. ZSM-5 in sodium and acid form (NaZSM-5 and HZSM-5) CR and CRR: biomass–PVC mixture varying the biomass/PVC–zeolite mass ratio (2:1, 1:1 and 1:2)	I: CDS Pyro probe 6200 T: 650 °C HR: 20 °C/ms t: 20 s.	I: Agilent Technologies 7890B/5977B GC/MS CC: Elite-35MS capillary column (30 m × 0.25 µm) T: 40 to 280 °C HR: 5 °C/min SR: 1:80	➢ The mineral content in the catalysts → ↑ mono-AHs ➢ ↓ poly-AHs ➢ 1:2 ratio of OP/PVC-HZ or AS/PVC-HZ blend → ↑ BTX, toluene and xylene	The use of HZMS-5 catalysts during co-pyrolysis leads to a reduction in acid and an increase in AHs through a series of reactions such as deoxygenation, isomerization, and oligomerization. Additionally, the formation of toluene and xylene is enhanced through the processes of dehydration and demethoxylation of phenolics.	[60]
Rice husk– main components of *Enteromorpha clathrata* (protein, polysaccharide, and ash)	ZSM-5 and MCM-41 CR and CCR: 1:1:1:1 (rice husk:seaweed component: catalyst)	I: CDS 5250 Pyro Probe, Oxford, PA, USA T: 550 °C HR: 10,000 °C/s CG: Ar at 1	I: Agilent Technologies 7890 A/5975C CC: HP-5MS—30 m × 250 μm × 0.25 μm T: 40 to 200 °C at 5 °C/min, and raised to 280 °C at 10 °C/min SR: 80:1 SC: 35 to 550 m/z	➢ Blends of both catalysts → ↓ oxygenated compounds and ↑ mono-AHs	Free radicals in the biomass result in AH compounds due to cracking, reformation, oxidation, and polymerization. ZSM-5 favoured dehydration, decarboxylation, cracking, and aromatization reactions to remove oxygenated compounds. MCM- 41 decreased oxygenates and increased aliphatics and AHs.	[61]
Polysaccharides–CE BR: 1:1	MCM-41, ZSM-5 CR: 1:1	I: Py: CDS5200, CDS Analytical Co. Ltd. T: 550 °C HR: 20 °C/ms t: 20 s CG: He	I: Agilent Technology, 7890A/5975i CC: HP-5 ms T: 280 and 300 °C SC: 35 and 550 amu	➢ Co-pyrolysis → synergistic effect Catalysts → ➢ ↑ 5-methyl-2-furaldehyde and 2-furaldehyde ➢ ↓ formation of anhydrosugars	DFT calculation → free radicals pyrolysed from soluble polysaccharides → **↓** ring-opening reaction of D-glucopyranose.	[62]
*Spirulina*–oil shale BR: 0, 10, 30, 50, 70, 90 and 100 wt.% of SP	HZSM-5 and CaO CR: 1:1 (BR was set at 1:1) CRR: 1:0, 3:1, 1:1, 1:3 CaO/HZM-5 ratio	I: Pyro probe 5200 Pyrolyser (CDS Analytical) T: 600 °C HR: (20 °C/ms) t: 20 s CG: N_2_	I: 7890A/5975C GC/MS analyser T: 50 to 290 °C HR: 10 °C/min SR: 1:50 SC: 35–300 amu CG: He at 3	➢ HZSM-5 and CaO → ↑ deoxygenation and aromatization reaction, acids, and phenols ➢ ↑ of CaO ratio → ↑ aliphatic hydrocarbons from 9.57% to 17.48%; AHs ↑ then ↓ → maximum yield → 15.92% was obtained at 1 CaO/3HZSM-5	CaO →↑ secondary cracking reaction, **↓** coke deposition. Combined use of catalysts since HZSM-5 →↑ hydrocarbon production via cracking, dehydration, decarbonylation and aromatization reactions.	[63]
Bamboo residual–waste tire	1 mg each, separated by quartz wool → HZSM-5, CaO, mixture of feed, CaO, HZSM-5.	I: CDS Pyro probe 5200 Pyrolyser T: 600 °C HR: 2000 T: 600 °C/s CG: He at 1	I: GC/MS, 7890A/ 5975C, Agilent CC: HP-5MS, 0.25 mm × 0.25 μm × 30 m SR: 1:80	➢ Catalysts in succession → ↑ AHs and olefins and ↓ acids ➢ Highest hydrocarbon yield at HZSM-5 to CaO mass was 3:2	Combination → effective removal of acids and improved formation of AHs and olefins through catalytic cracking, neutralization, and thermal cracking. Zeolites provided shape selectivity for deoxygenation and increased hydrocarbon yields.	[64]
Bamboo residual–waste lubricating oil (WLO)	Dual catalytic beds of MgO and HZSM-5 CR: 1:2	I: CDS analytical Pyro probe 5200 Pyrolyser T: 500–700 °C HR: 2000 °C/s CG: He	I: GC/MS, 7890A/5975C, Agilent CC: HP-5MS, 0.25 mm × 0.25 μm × 30 m T: 40 to 180 °C at 5 °C/min, and then to 280 °C at a 10 °C/min SR: 1:80 SC: 28–350 m/z	➢ MgO →↑ phenols selectivity ➢ HZSM-5 and MgO in a 3:2 ratio → highest AHs ➢ Addition of WLO → ↑ AHs, olefins, and alkanes	MgO exhibited more deacidification and ketonisation. HZSM-5/MgO highest yields of AHs via Diels–Alder reaction. WLO increases the yields due to hydrocarbon pool reactions.	[65]

**Table 3:** Findings of catalysts other than zeolites from selected articles reviewed in this study.

Feedstock	Catalysts	Pyrolysis Operating Conditions	GC-MS Operating Conditions	Products	Reaction Mechanism	Reference
Beechwood (BW) and red mud (catalyst) (RM) BR: BW: RM 1:1, 1:2, 1:4	Oxides: α-Al_2_O_3_, Fe_2_O_3_, and SiO_2_ in red mud as catalyst	I: 5200 Pyrolyser (CDS Analytical, Oxford, PA, USA T: 500 °C HR: 20 °C/ms t: 20 s CG: He at 30	I: 7890A/5975C gas chromatograph (GC)/mass spectrometer (MS) (Agilent Technology, Santa Clara, CA, USA) CC: HP5MS T: 50 to 300 °C SR: 1:50 SC: 35–400 amu	➢ Red mud → **↓** phenols ➢ Optimum: BW/RM-950 (1:4) ➢ **↓** Fe and Al in RM-950 → **↓** products than pure metal oxides	RM-950, Fe_2_O_3_, and TiO_2_ during depolymerization of CE and hemicellulose → increased furfurals and acetic acid→ increased depolymerization reactions and selectivity towards the products.	[78]
Textile dyeing Sludge (catalyst) –cattle manure BR: 0.9:0.1, 0.7:0.3, 1:1, 0.3:0.7, 0.1:0.9	Iron in the feed as a catalyst	I: Pyrolysis reactor (Frontier Lab PY-2020id, Fukushima, Japan) t: 24 s CG: He	I: Thermo DSQ Ⅱ, USA Capillary column (CC): HP-5MS T: Started at 45 °C for 2 min, increased to 300 °C at 4 °C/min, and remained at 300 °C for 15 min	➢ Co-pyrolysis at high temperatures → ↑ alcohols, phenols, ethers, aldehydes, ketones, and carboxylic acids ➢ ↑ G-type compounds in the volatile products	During the co-pyrolysis of the blend, the mechanism of the process changed from a diffusion model to a reaction-order mode. Gases were primarily produced as a result of the diffusion reaction.	[79]
Microalgae–oil shale (catalyst) BR: OS of 1%, 3%, 7%, and 10%	OS as a catalyst	I: 5200 Pyrolyser (CDS Analytical, Oxford, PA, USA T: 500, 600, 700 and 800 °C HR: 20 °C/ms t: 20 s CG: Helium at 30 mL/min	I: 7890A/5975C gas chromatograph (GC)/mass spectrometer (MS) (Agilent Technology, Santa Clara, CA, USA) CC: HP5MS T: 50 to 290 °C SC: 50–300 amu	➢ Optimum: 600 °C → ↑ AHs and aliphatics ➢ **↓** acetic acid and alcohol yields ➢ ↑ anti-coke ➢ **↓** poly-AHs	Higher temperature—large molecules cleaved and cracked. High H/C ratio in OS → hydrocarbons and olefins → Diels–Alder reaction → AHs through cycloaddition. Chain scission of organic volatile matter during pyrolysis of OS due to high activated energy of the oxygenates in MA.	[80]
*Chlorella vulgaris*—kitchen waste BR: 1:0, 0.8:0.2, 0.5:0.5, 02:08, 0:10	CaCO_3_, CaO, SiO_2_, and permutite. CR: 100, 80, 50, 20 and 0 wt.%.	I: CDS5200, CDS Analytical Co. Ltd. T: 700 °C HR: 20 °C/ms t: 20 s CG: Helium	I: Agilent Technology, 7890A/5975i CC: HP-5 ms T: First: 50 °C for 2 min; second: to 250 °C for 5 min at a heating rate of 10 °C/min SR: 1:50 SC: 33 and 500 amu	➢ *C. vulgaris* and kitchen waste → ↑ hydrocarbons, BTEXs, alcohols and ketones ➢ aldehydes → **↓** N-containing compounds ➢ Better products when CaO → **↓** acids ↑ hydrocarbons ➢ Optimum: 50:50 blending ratio	Potential: chain breaking, group rearrangement, depolymerization, polymerization, dehydration, cyclization, isomerization, etc.	[81]
CE and high-density polyethylene BR: 1:1	AAEMs impregnated with potassium concentrations of 0.1, 0.14, and 0.4 M	I: 5200 Pyrolyser (CDS Analytical, Oxford, PA, USA T: 550 °C HR: 20 °C/ms t: 30 s CG: Helium at 1	I: 6890N/5973i gas chromatograph (GC)/mass spectrometer (MS) (Agilent Technology, Santa Clara, CA, USA) CC: Agilent DB-17 ms T: First: held at 40 °C for 4 min; second: ramped at 5 °C/min to the temperature of 230 °C; final: 2 min SR: -50:1 SC: 40–500 amu	➢ Yield of anhydrosugars and hydrocarbons **↓** with ↑ in K (catalyst) → negative synergistic effect	Co-pyrolysis → synergistic effect → promotes glycosidic bond cleavage and hydrogen transfer reactions → enhanced anhydrosugars and hydrocarbons. AAEMs during co-pyrolysis → furans and ketones through ring cracking and dehydration reactions of CE.	[82]
Strong-acid cation exchange resin (Amberlyst-15, Check sp A15)—CE	Sulfonic acid groups from A15 as the catalyst	I: 5200 Pyrolyser (CDS Analytical, USA T: 300 °C, 400 °C, 500 °C, 600 °C and 700 °C HR: 20 °C/ms t: 20 s CG: He at 1	I: GC/MS (7890A/5975C inert, Agilent Technology, USA) CC: Agilent J&W DB-1701 T: First: held at 50 °C for 3 min; second: increased to 280 °C at a rate of 5 °C/min; final: stayed at 280 °C for another 10 min	➢ ↑ T ↑ HR → AHs ➢ Below 400 °C → ↑ levoglucosenone	Sulfonic groups improved levoglucosenone and inorganic sulphur-containing molecules at temperatures below 400 °C → promotion of CE pyrolysis.	[83]

## Data Availability

Not applicable.

## Abbreviations

AAEMs	Alkali and alkaline earth metals
AHs	Aromatics
BTEX	Benzene, toluene, ethylbenzene, and xylene
BTX	Benzene, toluene, and xylene
CE	Cellulose
e-PCB	Epoxy-printed circuit board
FTIR	Fourier-transform infrared spectroscopy
HDPE	High-density polyethylene
LDPE	Low-density polyethylene
LLDPE	Linear low-density polyethylene
MSW	Municipal solid waste
NMR	Nuclear magnetic resonance
PE	Polyethylene
PP	Polypropylene
PVC	Polyvinyl chloride
Py-GC/MS	Gas chromatography–mass spectrometry
TGA	Thermogravimetric analysis
TMR	Tandem microreactor
